# In pediatric rheumatologic disease, methotrexate leads to mildly changed bloodwork on the second day after administration

**DOI:** 10.1186/s12969-022-00685-8

**Published:** 2022-04-07

**Authors:** Boris Hugle, Nadine Fischer, Johannes-Peter Haas

**Affiliations:** German Center for Pediatric Rheumatology, Garmisch-Partenkirchen, Gehfeldstrasse 24, 82467 Garmisch-Partenkirchen, Bavaria Germany

Methotrexate (MTX) is the most commonly used DMARD in the treatment of juvenile idiopathic arthritis (JIA) [1]. Recommendations regarding MTX monitoring recommend measurement of aspartate aminotransferase (GOT), alanine aminotransferase (GPT) and differential blood count [2, 3]. MTX is given as a single weekly dose creating a serum drug level during the following approximate 24 h [4]. There have been concerns that blood work taken during that time would show a transient increase in blood parameters, especially transaminases [5]. We performed a retrospective study on levels of transaminases and blood counts comparing them by the number of days following MTX administration.

Nine hundred seventy-four laboratory sample results from 445 patients with pediatric rheumatologic diseases (79.5% with JIA, 20.5% with other diseases) admitted to our centre between 2018 and 2021 and treated with MTX orally or subcutaneously for at least 3 months were extracted from the database of the German Center of Pediatric Rheumatology. Weekday of blood sampling and last methotrexate dose was determined to calculate the time difference in days. Laboratory values for GOT, GPT, lymphocyte and neutrophil count were determined and normalized. Statistical analysis using analysis of variance of the time difference between MTX and laboratory sampling of these four parameters was performed, as well as Chi-square analysis for values above the normal limit for GOT and GPT, and below the normal limit for lymphocytes and neutrophils.

A one-way ANOVA revealed that there was a statistically significant difference for GOT (F(6, 966) = 8.535, *p* < 0.0001), GPT (F(6,966) = 3.657, *p* = 0.001) and neutrophil count (F(6,966) = 4.841, p < 0.0001) in days of difference, with the highest/lowest values on day 2 after adminstration of MTX (Fig. 1). There was no statistically significant difference for lymphocyte count (*p* = 0.634). However, abnormal values were not found significantly more frequently on any day for GOT (*p* = 0.708), GPT (*p* = 0.243), lymphocytes (*p* = 0.566) and neutrophils (*p* = 0.368).

**Fig. 1:**
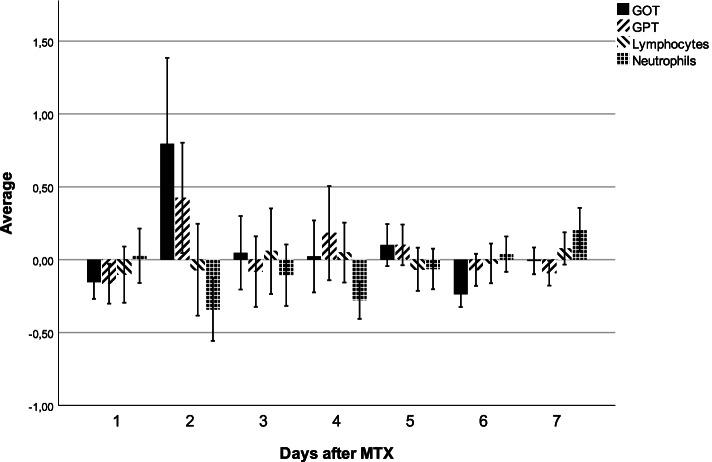
Normalized laboratory values of 974 blood samples from patients with pediatric rheumatic diseases according to their time in days after administration of methotrexate, mean value and standard deviation

MTX in the treatment of pediatric rheumatic diseases requires regular monitoring of blood parameters [2]. Changes in the levels of transaminases within a single week during therapy with MTX have already been investigated in a small study on 13 patients with rheumatoid arthritis, where the authors did not observe any significant change [5]. In this larger cohort of children with pediatric rheumatic disease we do find significant change, but not on the first, but rather the second day after administration. In the authors opinion, this effect is too small to merit any clinical note of caution. It is, however, of some value to the physician who observes elevated transaminases or depressed neutrophil counts 2 days after MTX administration.

## Data Availability

The datasets used and/or analyzed during the current study are available from the corresponding author on reasonable request.

## Abbreviations

ANOVA: Analysis of Variance
GOT: Aspartate Aminotransferase
GPT: Alanine Aminotransferase
MTX: Methotrexate
